# From snoRNA to miRNA: Dual function regulatory non-coding RNAs

**DOI:** 10.1016/j.biochi.2011.05.026

**Published:** 2011-11

**Authors:** Michelle S. Scott, Motoharu Ono

**Affiliations:** a Division of Biological Chemistry and Drug Discovery, College of Life Sciences, University of Dundee, Dow Street, Dundee DD1 5EH, UK; b Wellcome Trust Centre for Gene Regulation and Expression, College of Life Sciences, University of Dundee, Dow Street, Dundee DD1 5EH, UK

**Keywords:** Small nucleolar RNA, Micro RNA, Evolution, Dual function

## Abstract

Small nucleolar RNAs (snoRNAs) are an ancient class of small non-coding RNAs present in all eukaryotes and a subset of archaea that carry out a fundamental role in the modification and processing of ribosomal RNA. In recent years, however, a large proportion of snoRNAs have been found to be further processed into smaller molecules, some of which display different functionality. In parallel, several studies have uncovered extensive similarities between snoRNAs and other types of small non-coding RNAs, and in particular microRNAs. Here, we explore the extent of the relationship between these types of non-coding RNA and the possible underlying evolutionary forces that shaped this subset of the current non-coding RNA landscape.

## Highlights

► snoRNAs and miRNAs are extensively characterized small non-coding regulatory RNAs. ► snoRNAs and miRNAs have distinct and central regulatory roles in cells. ► recent studies reveal that snoRNAs and miRNAs display similarities at numerous levels. ► molecules showing both snoRNA and miRNA characteristics and functionality exist. ► subsets of snoRNAs and miRNAs likely share a functional and evolutionary relationship.

## 1 Small nucleolar RNAs, an ancient class of non-coding RNA

Small nucleolar RNAs (snoRNAs) are a large conserved group of abundant small non-coding RNAs predominantly serving as guides for the chemical modification of ribosomal RNA (rRNA) (reviewed in [1]). Over the past three years however, snoRNAs have attracted attention not only for their fundamental role in ribosome biogenesis but also because of their extensive processing and their likely involvement in other cellular regulatory roles. And while some snoRNAs may display only non-traditional functions, evidence is also accumulating of a subset of snoRNAs showing duality in their function.

Two main classes of snoRNAs have been defined, the box C/D snoRNAs and the box H/ACA snoRNAs, that differ in terms of sequence and structural elements, binding partners and nature of the chemical modification catalyzed. Both types of snoRNAs bind specific conserved protein partners, forming complexes referred to as snoRNPs (small nucleolar ribonucleoprotein complexes). Box C/D and H/ACA snoRNAs are highly conserved throughout evolution and are present in all eukaryotic organisms examined thus far. They have also been identified in some archaea where they are known as small RNAs (sRNAs) [2,3]. In addition, core snoRNP proteins are highly conserved in eukaryotes and have homologues in archaea, indicating that traditional snoRNP functions are ancient and fundamental [4,5].

Box C/D snoRNAs are molecules of approximately 60–200 nucleotides in length characterized by the presence of highly conserved boxes referred to as C (canonical motif RUGAUGA where R is a purine) and D (canonical motif CUGA). Box C/D snoRNPs carry out the 2′-O-ribose methylation of specific rRNA residues [6]. The boxes C and D, which are respectively found near the 5′ and 3′ ends of the molecule, come into close proximity in the folded molecule, forming a characteristic K-turn motif and serve as a binding site for core box C/D snoRNP proteins, including, indirectly, the methyltransferase fibrillarin [1,5]. A second pair of characteristic boxes, referred to as C′ and D′ are found closer to the middle of the molecule but display often lower sequence conservation than the boxes C and D [5]. The guide region, which ensures the specificity of the modification by base pairing to the target RNA, is a region of 10–20 nucleotides found immediately upstream of the boxes D and/or D′ [5,7].

Box H/ACA snoRNAs are typically longer than box C/D snoRNAs (approximately 120–250 nucleotides long) and serve as guides for the pseudouridylation of specific rRNA residues (reviewed in [1,6]). Box H/ACA snoRNAs generally fold to form two hairpins connected by a hinge region characterized by the presence of the box H (ANANNA where N can be any nucleotide). The second characteristic box, consisting of the trinucleotide ‘ACA’ is located three residues upstream of the 3′ end of the molecule, terminating the second hairpin. Internal stem-loops in one or often both hairpins serve as guides for the pseudouridylation of their substrates which is carried out by the pseudouridine transferase dyskerin, a core H/ACA snoRNP protein [5,6,8].

In addition to the two main types of snoRNA described above, a third class, the small Cajal body-specific RNAs (scaRNAs) has also been described. As their name implies, scaRNAs accumulate and function in Cajal bodies, small membrane-less sub-compartments of the nucleus, where they have mainly been found involved in the post-transcriptional modification of small nuclear RNAs (snRNAs). scaRNAs are larger than the predominant classes of snoRNAs and display characteristic boxes of both C/D and H/ACA snoRNAs as well as CAB boxes (motif UGAG) which function as a Cajal body localization signal [9].

In eukaryotic genomes, snoRNAs have been found both encoded in introns of protein-coding host genes as well as under the control of independent promoters [10]. In vertebrates, most snoRNAs are intronic and co-transcribed with the host gene transcripts, then processed out of the excised introns [11,12] but a small number are transcribed from either independent RNA polymerase II or III units [10,13]. The host genes of intronic snoRNAs are often involved in processes related to snoRNA function and more generally related to nucleolar function and protein synthesis (for example genes encoding snoRNP core proteins, ribosomal proteins and terminal oligo-pyrimidine (TOP) genes) although a number of snoRNA host genes do not appear to encode proteins [10,12]. Closely related snoRNA family members are often encoded in different introns of the same host gene, although some host genes encode several unrelated snoRNAs (even both box C/D and H/ACA snoRNAs within the same gene).

Although most snoRNAs are involved in the chemical modification of rRNA, a subset have been shown to target other substrates including small nuclear RNAs (snRNAs) or are involved in rRNA processing (reviewed in [1,6]). However, in addition to these roles related to classical snoRNA functions, numerous orphan snoRNAs have been described, with no known target [14,15], suggesting other classes of targets exist. And recent years have indeed been prolific in portraying snoRNAs under a new light.

## 2 Small RNAs derived from snoRNAs

In 2008, an unbiased sequencing study of human small RNAs (19–40 nucleotides) led to the observation of specific processing and the accumulation of small RNAs originating from well-characterized non-coding RNAs including snoRNAs [16]. While small RNAs were mapped to only a small number of snoRNAs, this study demonstrated that some snoRNAs are further processed to generate stably accumulating fragments. Later the same year, two groups reported snoRNA-derived molecules associating with Argonaute proteins and displaying microRNA (miRNA) capabilities [17,18]. Deep sequencing of human small RNAs associated with Argonaute proteins led to the identification of small fragments derived from the scaRNA ACA45. Further characterization revealed miRNA characteristics of the processed fragment, as well as additional human box H/ACA snoRNAs and scaRNAs displaying this processing signature [17]. In parallel, a *Giardia lamblia* box C/D snoRNA was also found to be processed into a smaller molecule with miRNA capabilities and characteristics [18].

The following year, snoRNA processing was shown to be widespread and conserved, through the description of snoRNA-derived small RNAs (termed sdRNAs by the authors) originating from a large number of snoRNAs in animal, plant and yeast genomes [19]. Since then, several reports have described other snoRNAs processed to generate smaller fragments with miRNA-like functionality, as well as miRNAs and snoRNAs that co-localize in the genome and known miRNAs with snoRNA-like features [20–23]. In addition, several other characteristics of these two families of small RNAs display great similarity, as discussed below.

## 3 miRNAs, post-transcriptional regulators of translation

Initially discovered in *C. elegans* almost twenty years ago [24], mature miRNAs are ∼23 nucleotide-long RNAs that are widely expressed in animals and plants and are processed out of precursors of variable length (typically 70 nucleotide-long hairpins in animals, but up to 300 nucleotide-long in plants) [25–28]. In human, approximately 60% of miRNAs are encoded in introns [10,29], of both protein-coding and non-protein-coding genes [30]. Intronic miRNAs are often co-expressed with their host genes and excised out of introns [30–32]. Other miRNAs are encoded in independent transcription units, some of which are under the control of the RNA polymerase II [33] while others are transcribed by the RNA polymerase III [34]. Primary miRNA transcripts are cleaved by Drosha, a nuclear RNase of type III, generating the miRNA precursor hairpin [32,35] which is then exported to the cytoplasm and further processed by the cytoplasmic RNase III enzyme Dicer, generating the mature miRNA [36–38]. However, numerous alternative pathways differing from the canonical miRNA biogenesis pathway have been described recently and subsets of several diverse longer non-coding RNAs can serve as precursors for miRNAs (reviewed in [39]).

Mature miRNAs are complexed to Argonaute proteins [27,40], forming an RNP complex involved in translation regulation through base pairing with its targets. In animals, mature miRNAs often incompletely base pair with regions in the 3′UTR of their targets and trigger deadenylation of the mRNA followed either by maintenance in a translation repressed state or decapping and 5′-to-3′ decay [41–43]. However, a small number of miRNAs have also been reported to activate translation [44]. In plants, mature miRNAs are typically highly complementary to regions mainly in the coding sequence of their targets and often instigate target cleavage [28,45].

## 4 Similarities between snoRNAs and miRNAs

### 4.1 Processing pathways and binding partners

As observed by Mattick in 2003, intronic snoRNAs and miRNAs utilize a similar set of processing enzymes to progress from encoded in an intron in a pre-mRNA, to the fully functional small RNA molecule [46]. Although several different processing pathways and sets of enzymes have been described for the processing of both snoRNAs and miRNAs, subsets of both types require the exosome functionality, followed by the action of exo and endonucleases [11,47,48]. And after processing out of introns, polycistronic snoRNAs in plant and yeast require the activity of the RNA endonuclease Rnt1 (the yeast ortholog of bacterial RNase III) to generate the individual molecules [48,49] while miRNAs are further processed by two RNase III enzymes, Drosha and Dicer as described above. In addition, as discussed above, Dicer has been shown to accept some snoRNAs as substrates including the human scaRNA ACA45 [17] and the Giardia lamblia box C/D snoRNA GlsR17 [18]. Thus the processing steps of subsets of snoRNAs and miRNAs (in particular the intronic members of these families) show great similarities and might share some components or be influenced by cross-talk between these related pathways.

In addition to similarities in processing pathways, unrelated observations have shown relationships between the protein interaction partners of snoRNAs and miRNAs. The box C/D snoRNP component fibrillarin responsible for the methyltransferase activity of the complex has been identified in AGO2 complexes and another box C/D snoRNP core protein, NOP56, in AGO1 complexes [50,51]. AGO1 and AGO2 are Argonaute proteins, known binding partners of miRNAs and members of the RNA-induced silencing complex (RISC) which carries out the silencing function in complex with miRNAs [52]. In addition, various Argonaute proteins have been found to associate with smaller fragments derived from snoRNAs, including AGO7 in Arabidopsis and Ago1 in Schizosaccharomyces pombe [19]. In human, as mentioned above, several box H/ACA snoRNA-derived fragments were identified in AGO complexes [17] and more recently, several processed snoRNAs were shown incorporated into RISC complexes [20].

### 4.2 Subcellular localization

In addition to a partial overlap and interaction of snoRNA and miRNA processing enzymes and functional binding partners, the subcellular localizations of many of these proteins and a subset of these RNA species also display commonalities between miRNAs and snoRNAs. In mammals, the miRNA processing enzyme Drosha has been detected in the nucleolus [53–55] and Dicer was found to encode several cryptic nuclear localization signals [56]. Moreover, while processing of miRNA precursors and release of mature miRNAs is believed to take place in the cytoplasm, a large proportion of human mature miRNAs have been detected in the nucleus, some accumulating predominantly in this compartment [57]. In addition, many mammalian mature miRNAs as well as miRNA precursors have specifically been detected in the nucleolus, subsets of which accumulate strongly in this compartment [21–23]. Conversely, a subset of small RNAs derived from snoRNAs have been detected in the cytoplasm in *G. lamblia* [18] and human [20].

### 4.3 Genomic organization and location

Similarity in the genomic organization and location of snoRNAs and miRNAs is striking. As described above, both groups have members encoded in introns and others encoded in independent transcription units [46,48]. Furthermore, the proportion of intronic miRNAs generally correlates with the proportion of intronic snoRNAs across numerous organisms. So while the majority of both snoRNAs and miRNAs are intronic in most vertebrates, most are encoded in independent transcription units in plants [48]. In addition, both snoRNAs and miRNAs can be found clustered in genomes although the positional nature of the clusters (whether intronic or in independent transcription units) is not highly correlated for miRNAs and snoRNAs [10,48]. In mammals, several examples exist of protein-coding host genes that encode both snoRNAs and miRNAs in distinct introns (for example the human serotonin receptor 2C (HTR2C) gene encodes the box H/ACA snoRNA HBI-36 as well as the miRNAs miR-448, miR-1264, miR-1298, miR-1911 and miR-1912 and the gene encoding the box C/D snoRNP core protein NOP56 also encodes the snoRNAs HBII-55, ACA51, U86, U56, U57 and the miRNA miR-1292).

### 4.4 Duplication and evolution

Members of both the snoRNA and miRNA classes show high levels of conservation [58–60]. snoRNAs are believed to be the most ancient, with members found throughout Eukaryota as well as in a subset of Archaea [2]. miRNAs have not been traced to such distant organisms although several families are well conserved in metazoans [60,61]. In contrast however, numerous studies also show the existence of recently evolved snoRNA and miRNA members that only exist in one or a small number of organisms [60,62,63], suggesting these families are dynamic and evolving at a fast rate. For both snoRNAs and miRNAs, many of the most recently evolved members are believed to be derived from transposable elements (TEs).

TEs are highly abundant elements in many mammalian and plant genomes which have the capacity of moving or copying themselves from one genomic site to another [64,65]. Through computational searches and sequence analyses, several groups have identified hundreds of human snoRNAs and miRNAs displaying characteristics and sequence elements of TEs suggesting these molecules were derived from retroposition of existing molecules that were inserted into new genomic locations [34,58,62,63,66–69]. Many such human snoRNA-like molecules, which were named snoRTs (snoRNA retrogenes), have been identified surrounded by retrogene features including flanking target site duplications, L1 consensus recognition sites and 3′ end poly (A) tails suggesting they used long interspersed nuclear element (LINE) machinery for retroposition [62,63]. Consistent with this, several snoRNAs have been found to change their host genes in the course of evolution, resulting in orthologous snoRNAs residing in different host genes when different organisms are compared [70,71]. While some snoRTs are likely non-functional, others were reported as functional snoRNAs [62]. Similarly, hundreds of miRNAs have also been identified encoded within TEs, of both LINE and SINE (short interspersed nuclear element) types [34,66,67]. These copies of snoRNAs and miRNAs might serve to safeguard against mutations in parental molecules. In addition, however, because they are under less evolutionary pressure to remain intact, being redundant, they likely also lead to the rapid evolution of snoRNAs and miRNAs with new targets [63,67,68,72].

## 5 Evolutionary intermediate versus dual function molecule

The similarity on numerous levels of snoRNAs and miRNAs offers new evolutionary hypotheses to contemplate. While this similarity might be the result of convergent evolution due to the availability of duplication mechanisms and processing enzymes in the cell, it might also stem from evolution from a common ancestral RNA molecule or evolution from one small RNA type to the other.

**Fig. 1:**
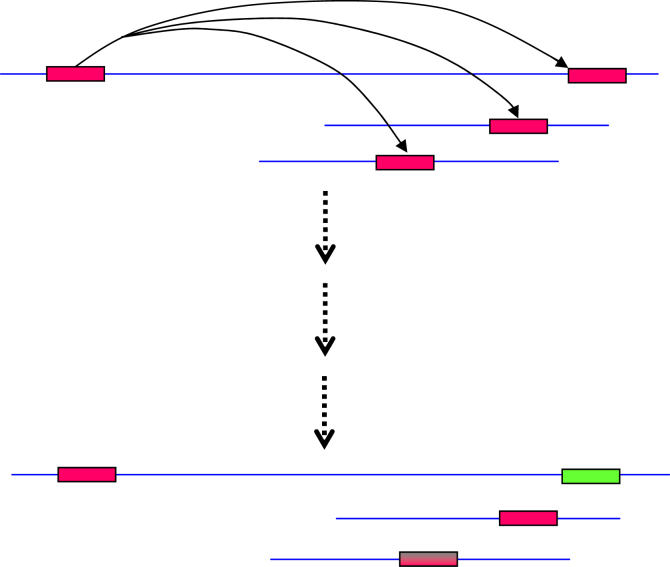
Proposed hypothesis for the evolution of snoRNAs. snoRNAs have been described as mobile genetic elements that can copy themselves to other genomic locations, whether on the same or different chromosomes [62,63]. These snoRNA retrogenes and their parental copies are subjected to evolutionary forces and might either retain snoRNA functionality (if they do not gain deleterious mutations and are in the right genomic context), become inactive (due to deleterious mutations or improper expression) or gain new functionality. Here, chromosomes are represented by blue lines, snoRNAs by pink boxes, mutated snoRNAs by pink and grey boxes and non-coding RNA elements with new functionality with green boxes.

The extent of stable association of snoRNAs and miRNAs with their host genes was investigated in a very insightful study [73] and might provide clues to address this question. The authors found that 21% of snoRNAs and 11% of miRNAs can be traced back to the mammalian ancestor and are referred to as ancestral positional conserved (APC). APC snoRNA host genes are highly over-represented in the cellular processes of translation and ribosome biogenesis while other (non-APC) snoRNA host genes show no such strong enrichment suggesting an ongoing functional diversification of snoRNAs from their ancestral role in the biogenesis of ribosomes [73]. Interestingly, the authors found no such functional association for APC or other miRNA host genes. This analysis and the ancient lineage of snoRNAs which likely arose over 2–3 billion years ago [1] allows the formulation of the hypothesis that when snoRNAs started diversifying by copying themselves to other genomic locations and likely acquiring new targets, a subset progressively lost snoRNA functionality and gained new (and possibly miRNA) capabilities (illustrated in Fig. 1).

In support of this hypothesis, miRNA precursors displaying snoRNA characteristics on multiple levels from genomic context similarity to sequence and structural similarity have recently been reported [21,23]. Using computational approaches, 20 and 84 human miRNA precursors were identified showing characteristic boxes as well as regions of complementarity to rRNA and structural characteristics typical of H/ACA and C/D snoRNAs respectively. In the case of box H/ACA snoRNA-like miRNA precursors, five such molecules were further experimentally analysed showing H/ACA functionality through binding to dyskerin, a core H/ACA snoRNP protein [23]. Similarly, five miRNA precursors with box C/D snoRNA characteristics were found to bind to fibrillarin, a core box C/D snoRNP [21]. Interestingly, it was also recently found that the loss of dyskerin led to reduced levels of H/ACA snoRNA-derived miRNA-like molecules [74].

**Fig. 2:**
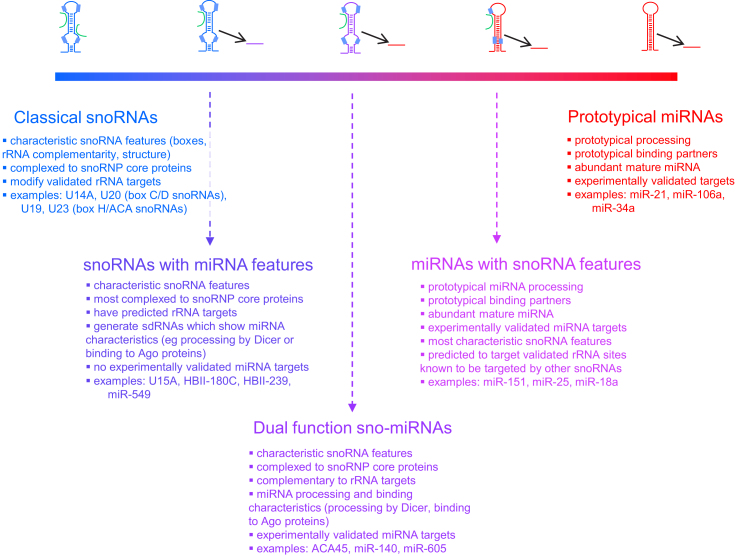
The snoRNA-miRNA spectrum in human. Through numerous independent studies, we now have many examples of small RNA molecules displaying both snoRNA and miRNA characteristics, suggesting a possible evolution from one type to the other. Here, the typical structure of box C/D snoRNAs (molecule shown in blue) is used to represent any type of snoRNA. Green lines represent complementary rRNA molecules. miRNAs (hairpin and mature fragment) are represented in red. Classical snoRNAs were chosen from their description in snoRNAbase [81]. SnoRNAs with miRNA features are described in [20,21,23,82]. The dual function sno-miRNAs were described in [17,23]. The snoRNA characteristics of the miRNAs with snoRNA features were described in [21,23] while their miRNA features are described in [83–85]. Human prototypical miRNAs were chosen from the list defined in [86] for which experimentally validated targets have been reported according to version 5c of TarBase [87]. (For interpretation of the references to colour in this figure legend, the reader is referred to the web version of this article.)

In the list of known miRNA precursors showing strong snoRNA characteristics, some molecules were actually poorly characterized as miRNAs. For example, the precursor of human miR-549 is predicted with high score to be a box H/ACA snoRNA. It displays the characteristic features and structure of a box H/ACA snoRNA, is predicted to target an experimentally validated rRNA pseudouridylation site for which no guide is known in human (and thus the miR-549 precursor is the best current candidate to fulfil this role in the human genome) and its mature miRNA was detected at a very low count [23,75], suggesting it might be better annotated as a snoRNA or could be at the beginning of the process of evolving into a miRNA. However, amongst the snoRNA-like miRNA precursors that display sequence, structural and functional snoRNA characteristics, there are well-characterized and validated miRNAs (eg let-7g, miR-140, miR-215 and miR-28 [21,23]), offering convincing examples of molecules that function both as snoRNAs and miRNAs. In addition, recent reports are providing strong support for the functions of both snoRNAs and miRNAs within the same molecule. For example, the precursor of human miR-605 was found to display strong box H/ACA snoRNA characteristics and structure, is predicted with high score to target an experimentally validated rRNA pseudouridylation site for which no guide is known in human and binds to dyskerin, a core snoRNP protein, suggesting it is actually a box H/ACA snoRNA [23]. However, miR-605 has also recently been found involved in the p53 network and its overexpression caused the post-transcriptional reduction of Mdm2 expression while its knockdown had the opposite effect, thus suggesting miRNA functionality [76]. We now have several such examples of dual function snoRNA-miRNAs, as well as snoRNAs with miRNA features and miRNAs with snoRNA features (illustrated in Fig. 2).

Another recent study provides evidence of specific accumulation of snoRNA-derived small RNAs (sdRNAs) in a physiological setting. The up-regulation of small RNAs derived from several types of non-coding RNAs was detected in the adult mouse hippocampus during olfactory discrimination training [77]. In particular, in mice trained by the use of rewards to recognize specific odours, but not in control animals, strong increases of approximately 100-fold of specific sdRNAs of size 25–30 nucleotides were detected. Although these sdRNAs might be involved in supporting protein synthesis during this task, the fact that not all snoRNAs with known rRNA targets accumulate at such a high level rather suggests that they might participate in alternative functions, possibly involved in learning [77]. Some of the snoRNAs from which small RNAs are derived in this context are well-characterized molecules with known rRNA targets, suggesting that these snoRNAs are indeed bi-functional.

Given the spectrum of molecules displaying snoRNA and miRNA characteristics (Fig. 2), it is interesting to consider where the orphan snoRNAs fit in. These molecules have typical snoRNA features (characteristic boxes and structure, binding to core snoRNP proteins) but they do not have a region of complementarity to classical snoRNA targets. Many orphan snoRNAs were found to generate sdRNAs and it has been suggested that they might function as intermediates in this pathway [19]. Many orphan snoRNAs are also known to be snoRNA retrogenes [62] and might represent copies of functional parental snoRNAs which have lost their guide region and acquired (or always had) the capability of serving as a substrate for the sdRNA pathway and might have the potential of evolving to gain miRNA functionality.

It should also be noted that sdRNAs with functional cellular roles do not all function in translation regulation. A large family of box C/D snoRNAs, the HBII-52s in human and MBII-52s in mouse, have been found processed into smaller RNAs and were shown to regulate splicing of several different transcripts including the serotonin receptor 2C [78,79]. Through computational searches, it was also found that other orphan snoRNAs have likely targets near alternative splice junctions [80] suggesting the regulation of splicing might be another common function carried out by snoRNAs or sdRNAs. As only orphan snoRNAs have been described involved in the regulation of splicing, such molecules would likely not be annotated as dual function although they might be described in this light in the future.

## 6 Future perspectives

Recent years have been rich in reports highlighting diverse new characteristics and functions for previously described small non-coding RNAs. Subsets of two such classes, snoRNAs and miRNAs, increasingly display overlapping and common features. Thanks to rapid improvements in sequencing technologies, the next few years will likely provide a deluge of information that can address the extent of this commonality and which molecules are involved, leading to a better classification and understanding of these groups.

The true dual function sno-miRNA molecules will provide interesting subjects to study. These regulatory molecules are themselves likely to be under the control of other layers of regulation to ensure the appropriate balance of the different species originating from these molecules in different cell types and physiological conditions. In addition to providing a fascinating puzzle to decipher, they will likely be of relevance for human health issues.
